# Laparoscopic Cholecystectomy in Situs Inversus Totalis with Stage 5 Chronic Kidney Disease: A Case Report

**DOI:** 10.31729/jnma.8301

**Published:** 2023-11-30

**Authors:** Sneha Raut, Yugal Limbu, Prashanta Pudasaini, Swagat Gongal, Dhiresh Kumar Maharjan

**Affiliations:** 1 Kathmandu Medical College and Teaching Hospital, Sinamangal, Kathmandu, Nepal; 2 Department of Gastrointestinal and General Surgery, Kathmandu Medical College and Teaching Hospital, Sinamangal, Kathmandu, Nepal; 3 Department of Anesthesiology, Kathmandu Medical College and Teaching Hospital, Sinamangal, Kathmandu, Nepal

**Keywords:** *case reports*, *cholecystectomy*, *situs inversus*

## Abstract

Situs inversus totalis is a rare congenital anomaly in which the abdominal and thoracic organs are transposed in a mirror image. Diagnosis and management of cholelithiasis in patients with situs inversus totalis pose a challenge due to the anatomical variation. A laparoscopic cholecystectomy in such a case can be technically challenging, especially for a right-handed surgeon. In this case report, we present a case of a 38-year-old male with symptomatic cholelithiasis in a chronic kidney disease stage five patient under maintenance hemodialysis planned for recipient renal transplant. A laparoscopic cholecystectomy considered the gold standard for symptomatic cholelithiasis was performed with a three-port technique. The technical challenges anticipated due to anatomical variation were managed by intraoperative modifications. In conclusion, laparoscopic cholecystectomy in patients with situs inversus totalis can be done with technical modifications and re-orientation of visual motor skills.

## INTRODUCTION

Situs inversus totalis is a rare autosomal recessive condition which is characterized by the transposition of thoracic and abdominal viscera through the sagittal plane, creating a mirror image of normal anatomical structures. Incidence is reportedly 1 in 5000-20,000 live births.^1^ The stomach, spleen, and major lobe of the liver and gall bladder are on the right hypochondriac area in the abdomen, the apex of the heart is on the right side of the thorax, the left lung is tri-lobed, and the right lung is bi-lobed, and nerves, blood vessels, and lymphatics are also inverted.^2^

## CASE REPORT

A 38-year-old male presented with backache and vomiting for two months. The vomiting was nonprojectile, non-bile or blood-stained, following meals and containing undigested food particles. The patient has a known case of hypertension under medication for one year and a known case of chronic kidney disease stage 5 under maintenance hemodialysis planned for recipient renal transplant. Elective cholecystectomy was planned for symptomatic cholelithiasis to prevent complications following renal transplant. The general examination was normal. The abdomen was soft, and an umbilical hernia was present with features of enterocele. The remainder of the abdominal examination was unremarkable. The cardiovascular system examination revealed an apex beat in the right 5^th^ intercostal space mid-clavicular line. Patients routine blood investigation revealed neutrophilia (neutrophils= 81%), lymphopenia (lymphocytes= 16%), low hemoglobin (hb= 10.1 gm%) and elevated serum creatinine (4.6 mg/dl). The thyroid function test and liver function test showed no abnormality. Chest X-ray revealed dextrocardia.

**Figure 1:**
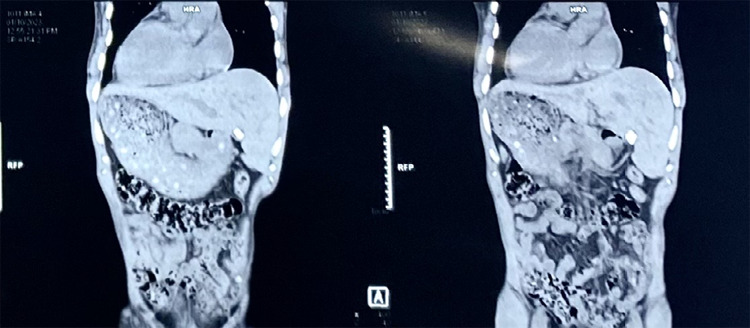
CT scan showing situs inversus totalis.

The ultrasonography (USG) of the abdomen revealed dextrocardia with visceral situs inversus, contracted gall bladder with cholelithiasis (multiple, largest measuring 19.3 mm) and umbilical hernia. The USG also revealed a right renal cortical cyst measuring 12.3 x 11.1 mm and grade four mild renal dysfunction of bilateral kidneys. The computed tomography scan (CT-scan) revealed total transposition of abdominal and thoracic viscera-situs inversus totalis, contracted gall bladder with cholelithiasis, small-sized bilateral kidneys with few cysts within the kidney on the left side of the abdominal cavity, minimal free fluid in the pelvic cavity and umbilical hernia (Figure 1).

The echocardiography showed dextrocardia; concentric left ventricular hypertrophy; left atrial enlargement; mild mitral valve regurgitation; no intracardiac mass, thrombus or pericardial effusion; normal left ventricular systolic function (50%).

A day before the surgery the patient underwent hemodialysis with 5000 units of heparin, ultrafiltrate measuring 2800 ml, pre-weight measured 49.6 kg and post-weight 47.4 kg. After clearance from anaesthesia, the patient was scheduled for elective laparoscopic cholecystectomy. On the operating table, the patient was positioned supine. An 18G intravenous cannula on right-hand-side was used to secure intravenous access, electrocardiography (ECG) electrodes (mirror image), non-invasive blood pressure and pulse oximeter were connected, and the patient had a radial arteriovenous fistula on the left hand so all apparatus were connected to the right hand. The operation theatre equipment arrangement was adjusted as a mirror image of routine laparoscopic cholecystectomy, the monitor was situated on the left side of the patient. Pre-oxygenation was done followed by modified rapid sequence induction (RSI) without using cricoid pressure with an injection of fentanyl, propofol, lignocaine and succinylcholine was given as coinduction. Atracurium injection was used to achieve neuromuscular blockade. The patient was intubated and anaesthesia was maintained with isoflurane in oxygen.

**Figure 2:**
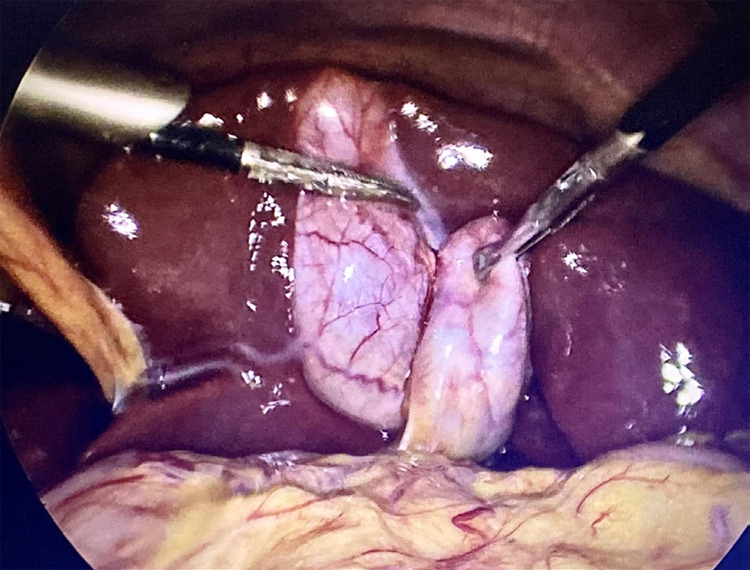
Gall bladder located to the left of the falciform ligament with Hartmann's pouch retraction.

The primary surgeon and first assistant were on the patient's right side. The abdomen was painted with betadine and draped in standard aseptic technique, a 10 mm-infra-umbilical port was created and pneumoperitoneum was achieved at 12 mm Hg followed by diagnostic laparoscopy confirming SIT and a 5 mm-left subcostal and 10 mm-epigastric ports were created under direct vision (Figure 2).

**Figure 3:**
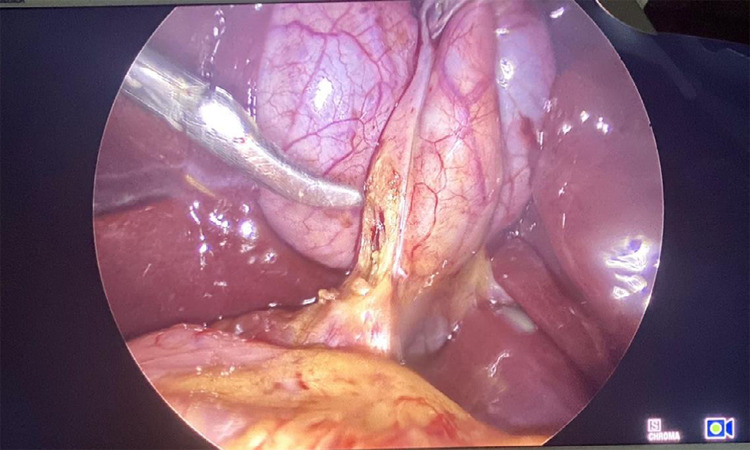
Calot's triangle dissection.

The dissection of Calot's triangle was carried out using a Maryland dissector on the right-hand port and an atraumatic bowel grasper on the left-hand port, critical view of safety was achieved (Figure 3).

The cystic artery and cystic duct were respectively clipped and divided. The gall bladder was dissected off the cystic plate, collected in an endoscopic bag and removed via infra-umbilical port after confirming hemostasis. There was minimal bile spillage and minimal bleeding. All port sites were closed with non-absorbable sutures and a specimen was sent for histopathology. On respiratory attempts by the patient, the residual neuromuscular blockade was reversed and the patient was extubated with regular oropharyngeal suctioning showing minimal secretions. The patient had an uneventful postoperative course and was discharged on the first postoperative day.

## DISCUSSION

This was a rare case of laparoscopic cholecystectomy in situs inversus totalis with stage five CKD done in our institution which required anaesthetic and surgical modifications to cater for the needs of the patient. Situs inversus is an autosomal recessive genetic disorder with a prevalence of 0.04% to 0.30% and an incidence of 1:5000 to 1:20000.^3^ There are two different types of situs inversus: situs inversus totalis (SIT), which involves both the thoracic organs and the abdominal viscera, and situs inversus partialis, which only affects the thoracic organs (dextrocardia).^4^

Fabricius reported the first known case of situs inversus in humans in 1600.^5^ It is linked to several other conditions, including Kartagener's (bronchiectasis, sinusitis, situs inversus) and cardiac anomalies.^5^ There is currently no evidence that situs inversus causes cholelithiasis.^5^ In a previous study, which was successfully performed as the first laparoscopic cholecystectomy in a patient with SIT in 1991.^4^ Since then, 92 cases have been reported in peer-reviewed journals.^4^ Although laparoscopic cholecystectomy is a safe procedure in SIT, the anatomical variation can bring about challenges, especially for a righthanded surgeon. A previous study described a four-port technique-a 10 mm umbilical port, a 10 mm-medial epigastric port and two lateral subcostal-5 mm ports.^6^ The surgeon used the epigastric port to retract Hartmann's pouch with the left hand and operate with the right hand via the lateral subcostal port.^6^ In another previous study, there was a description of a single incision laparoscopic surgery (SILS) in which a 2 cm infra-umbilical incision was used to insert the SILS port with three operating channels into the abdomen.^7^ Without any technical difficulties, Calot's triangle and the gallbladder bed were dissected using a dissector and hook in the right hand. A former study had a description of a four-port technique- a 10 mm infra-umbilical port, a 10 mm-epigastric port, a 5 mm-left hypochondrium port and a 5 mm-left lateral subcostal port.^8^ The first assistant retracted the Hartmann's pouch throughout the surgery while the main surgeon dissected the calot's triangle using his right hand from the epigastric port with convenience.^8^ However, we used the three-port method, using a 10 mm infra umbilical, 10 mm epigastric and 5 mm left subcostal which follows the "baseball diamond principle" of laparoscopic surgery ergonomics, keeping the manipulation angle at 60 degrees, the azimuth angle between 15 and 45 degrees and the elevation angle at 30 degrees. A similar technique was described in a previous study with a three-port approach carried out by a left-handed surgeon in which they placed a 10 mm infra umbilical camera port, a 10 mm epigastric port, and a 5 mm left subcostal port to successfully perform the laparoscopic cholecystectomy.^9^

The main challenge described in the previous cases was that the right-handed surgeon had to cross his hands to retract Hartmann's pouch while dissecting Calot's triangle. However, in our case, this challenge was overcome by our primary surgeon being ambidextrous. There were no major complications during the surgery. Since this is an end-stage renal disease patient, RSI was done and atracurium was used as it undergoes Hoffman degradation. When pneumoperitoneum was established, there was a minor complication in the form of bradycardia up to 56 beats/min which was overcome by injection of atropine. To optimise ergonomics and to help minimize the technical challenges, all the equipment and monitors in the operation theatre were arranged as the mirror image of the normal set-up.

The effective completion of laparoscopic cholecystectomy in SIT requires careful planning, reorientation of ergonomics and expert visual motor skills to adapt to the anatomical variance. With rigorous preoperative preparation and intraoperative modifications, the procedure can be completed safely.
